# Gastric adenocarcinoma at stage IV with complete remission after neoadjuvant therapy concurrent with adenosquamous carcinoma of the ampulla of Vater: a case report and literature review

**DOI:** 10.1186/s12893-021-01133-2

**Published:** 2021-04-30

**Authors:** Shuo Li, Mengqing Sun, Yingxin Wei, Yunlu Feng, Xiaoyan Chang, Yan You, Ziwen Liu, Xianlin Han

**Affiliations:** 1 Department of General Surgery, Peking Union Medical College Hospital, Chinese Academy of Medical Sciences, Beijing, 100730 China; 2 Department of Gastroenterology, Peking Union Medical College Hospital, Chinese Academy of Medical Sciences, Beijing, 100730 China; 3 Department of Pathology, Peking Union Medical College Hospital, Chinese Academy of Medical Sciences, Beijing, 100730 China

**Keywords:** Adenosquamous carcinoma (ASC), Ampulla of Vater (AmV), Gastric adenocarcinoma, Case report

## Abstract

**Background:**

Adenosquamous carcinoma (ASC) of the ampulla of Vater (AmV) is exceedingly rare with more aggressive behavior and worse prognosis than adenocarcinoma. The finding of ASC at the AmV in combination to the gastric adenocarcinoma has never been reported in the literature before.

**Case presentation:**

An old lady was diagnosed as gastric adenocarcinoma at stage IV with enlargement of supraclavicular lymph nodes by gastroscopy and histopathological evaluation 3 years ago. Afterwards, the patient achieved complete remission after regular chemotherapy. However, the patient manifested yellow sclera and skin, choluria and clay colored stool 3 months ago. Preoperative contrast-enhanced CT, ERCP, MRCP, and PET/CT revealed the presence of an ampullary tumor. The patient then underwent laparoscopic radical gastrectomy and pancreaticoduodenectomy with regional lymph node dissection. Postoperative cytological analyses confirmed the diagnosis of gastric ulcer with complete response to neoadjuvant therapy and ASC at the AmV. The patient’s postoperative outcome was uneventful.

**Conclusion:**

Drawing firm conclusions about the diagnosis of ampullary ASC is difficult because of the difficulty in acquiring both adenocarcinoma and SCC components by fine needle biopsy. The rarity of ASC of the AmV coexistent with gastric carcinoma makes it difficult to elucidate their clinicopathological characteristics, therapeutic strategies and overall prognosis. Surgical resection still remains the main treatment method.

## Background

Adenocarcinomas are the most common histological type of malignancies at the ampulla of Vater (AmV) [1]. Adenosquamous carcinoma (ASC) of AmV with both adenocarcinoma and squamous cell carcinoma components is exceedingly rare [1]. Combination of primary cancers at the AmV and stomach has already been very rare, while coexistence of ASC at the AmV and gastric adenocarcinoma, to the best of our knowledge, has never been reported. In this study, we report one patient with both gastric adenocarcinoma and ASC of AmV who was admitted to Peking Union Medical College Hospital. Written consent for publication of the patient's details including medical records and images has been obtained from the patient described in this study.

## Case presentation

A 67-year-old woman with a medical history of hypertension disease, chronic hepatitis and poorly differentiated gastric adenocarcinoma was referred to our hospital for investigation of decreased appetite and pruritus with no relevant findings in the abdominal examination for the previous 6 months.

**Fig. 1:**
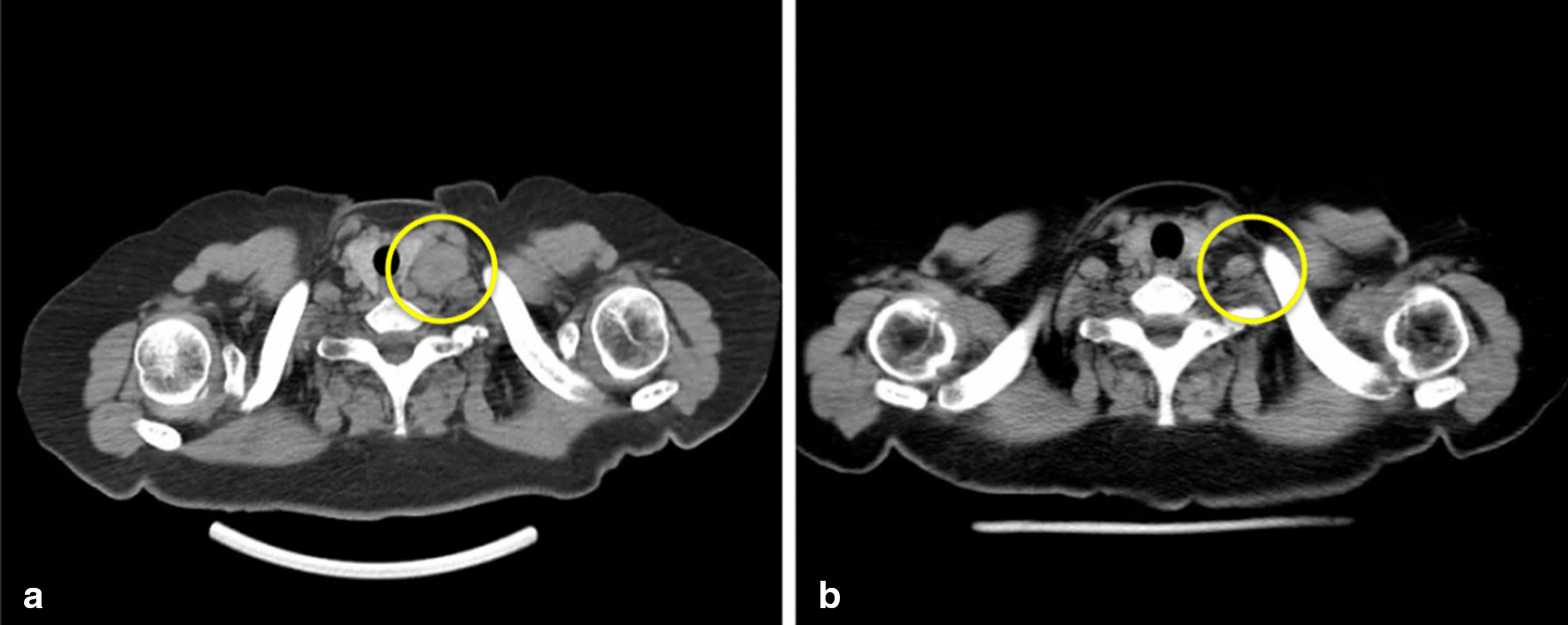
The contrast-enhanced CT showed the cervical lymph nodes on admission (**b**) significantly shrank compared with 3 months ago (**a**)

**Fig. 2:**
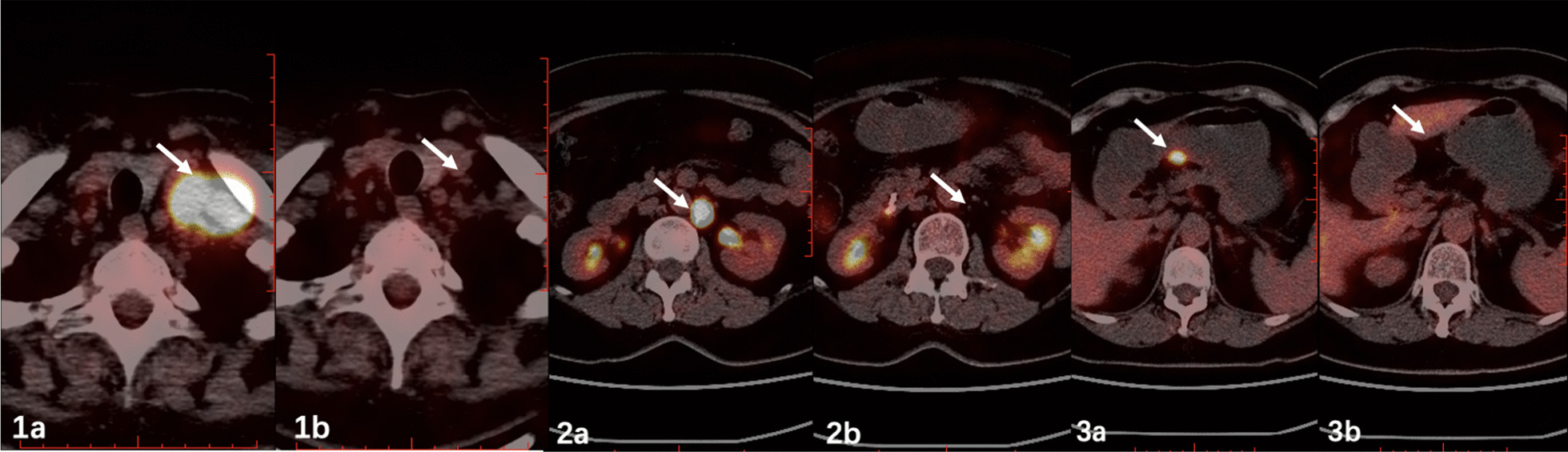
PET/CT revealed lymph nodes with increased metabolism at the bilateral supraclavicular fossa, para- abdominal aorta and lesser curvature of stomach on admission (Fig. 1b, 2b,3b) were no longer as obvious as 3 years ago (Fig. 1a, 2a,3a)

Gastroscopy performed 3 years ago revealed an obvious gastric mass around the gastric antrum which proved to be poorly differentiated adenocarcinoma by cytologic evaluation. The chest CT at the same time showed obvious enlargement of supraclavicular lymph nodes. Afterwards, the patient received 6-course chemotherapy of SOX (S-1 and oxaliplatin) regimen. Contrast-enhanced computed tomography on admission this time revealed the previously enlarged cervical lymph nodes significantly shrank (Fig. 1) and gastroscopy on admission also revealed the previous obvious gastric mass at the gastric antrum had shrank into an ulcer scar at the lesser curvature. Positron emission tomography/computed tomography (PET/CT) showed previous multiple lymph nodes with increased metabolism at the lesser curvature of stomach, para- abdominal aorta and bilateral supraclavicular fossa were no longer obvious on admission (Fig. 2). All of these evidences demonstrated that the patient’s gastric adenocarcinoma achieved complete response after neoadjuvant chemotherapy.

**Fig. 3:**
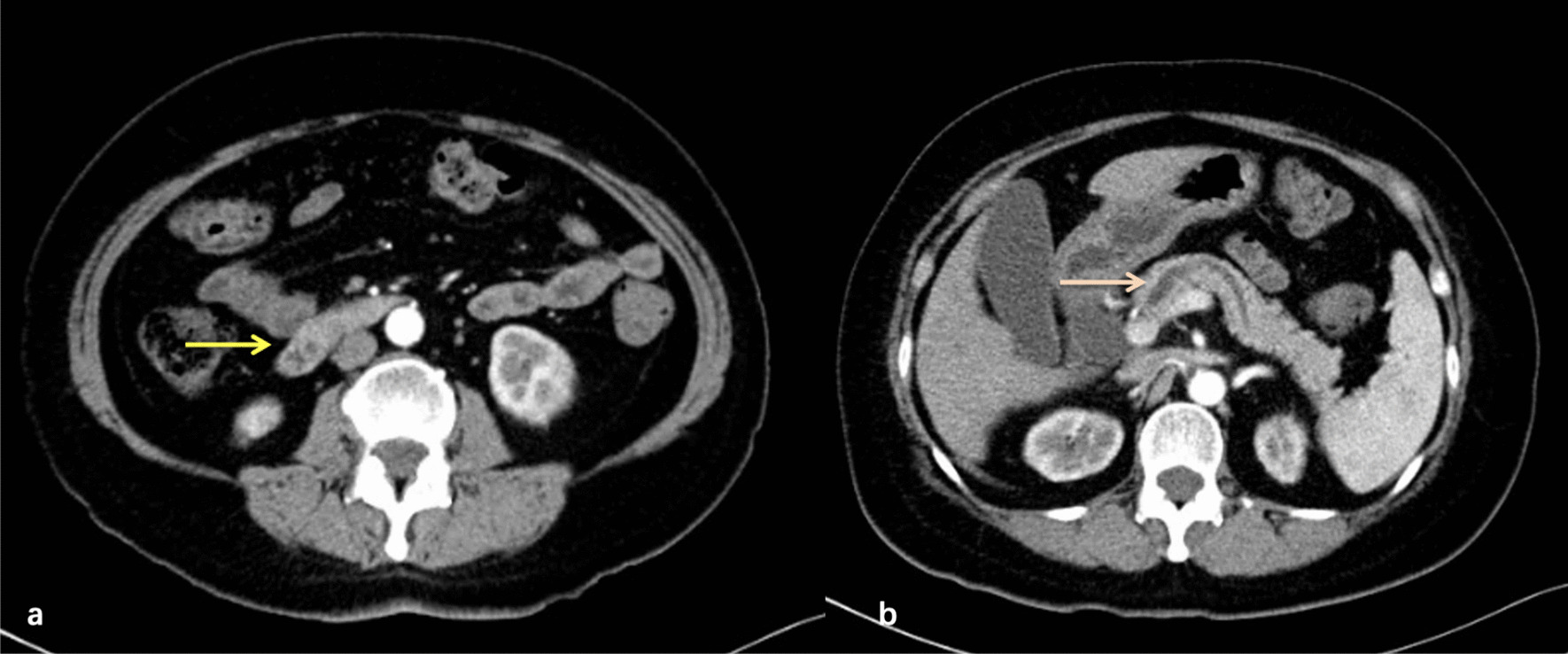
Contrast-enhanced CT 6 months ago found suspicious nodules at the duodenal papilla (**a**) and dilatation of pancreatic duct (**b**)

**Fig. 4:**
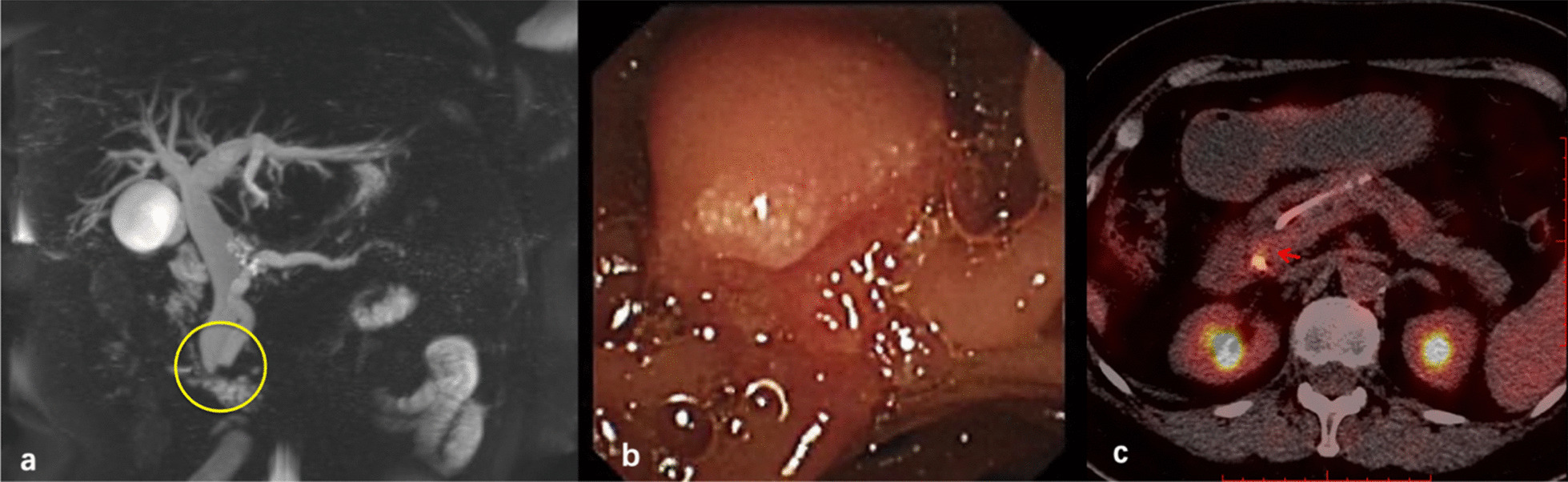
MRCP suggested the common bile duct and main pancreatic duct were suddenly narrowed at the duodenal ampulla (**a**). ERCP showed a bulging protrusion at the ampulla of Vater (**b**). PET/CT revealed an increased metabolic focus at the duodenal papilla area (**c**)

Contrast-enhanced abdominal computed tomography performed 6 months ago suggested dilatation of intra- and extrahepatic bile ducts and the pancreatic duct and the presence of suspicious nodules at the duodenal papilla (Fig. 3). Afterwards, she presented with yellow sclera and skin, choluria and clay colored stool that persisted for 3 months. Laboratory examinations revealed 64.6 μmol/L total bilirubin (normal, 5.1–22.2 μmol/L), 50.0 μmol/L direct bilirubin (normal, 0–6.8 μmol/L), elevated transaminase levels, including 38U/L alanine aminotransferase (normal, 7–40 U/L), 80U/L aspartate aminotransferase (normal, 13–35 U/L) and cholestasis parameters, including 1411U/L γ-glutamyl transpeptidase (normal, 7–45 U/L), 314U/L alkaline phosphatase (normal 50–135 U/L). Tumor markers carbohydrate antigen 19–9 was 58.1U/ml (normal, 0–34 U/ml)while levels of other tumor markers were within normal limits. Magnetic resonance cholangiopancreatography (MRCP) showed the common bile duct and main pancreatic duct were suddenly narrowed in the duodenal ampulla and the upstream bile duct was obviously dilated suggesting the presence of an ampullary tumor (Fig. 4a)**.** Neither Lymph node enlargement nor distant metastasis were found. Endoscopic retrograde cholangiopancreatography (ERCP) showed a bulging protrusion at the ampulla of Vater which was diagnosed on biopsy to be poorly differentiated adenocarcinoma (Fig. 4b). Implantation of bile duct and pancreatic duct plastic stent was performed during ERCP. PET/CT showed an increased metabolic focus of 1.0 × 0.8 cm and SUVmax 4.0 at the duodenal papilla area considered to be malignant lesions, the properties of which still needs to be determined (Fig. 4c). The patient has lost 8 kg for the last 3 months.

**Fig. 5:**
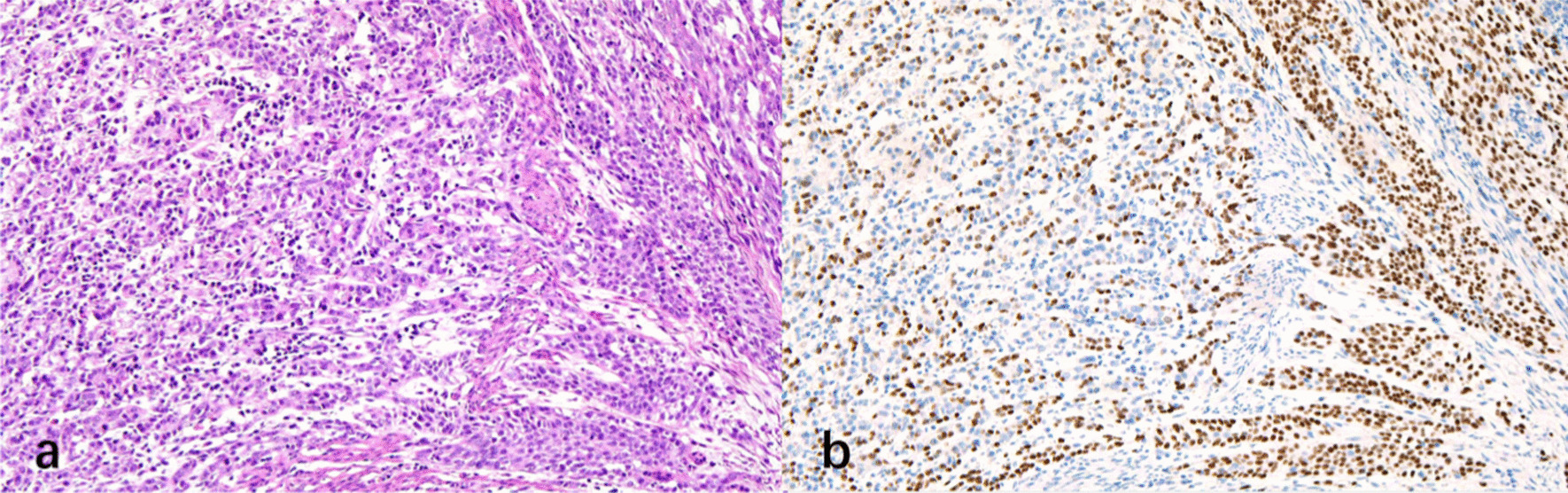
Pathological and immunohistochemical findings. Hematoxylin and eosin staining. CK -7 was detected in the adenocarcinoma component (**a**) and P 40 was positively expressed in the squamous cell carcinoma component (**b**) (original magnification ×200)

Levels of serum chemistry data and tumor markers on admission this time were within normal limits. Subsequently, laparoscopic radical gastrectomy and pancreaticoduodenectomy with regional lymph node dissection was performed. The gastric tumor was observed to be at the lesser curvature around the gastric antrum and was marked under the electronic gastroscopy during the operation. Pathological analyses documented the presence of adenosquamous carcinoma (ASC) at the ampulla of Vater with invasion into the duodenal muscular layer and gastric ulcer which is consistent with complete response to chemotherapy. No regional lymph node metastases or perineural infiltrations were observed. Immunohistochemistry examinations of the adenocarcinoma marker cytokeratin (CK)-7 showed positive expression in the adenocarcinoma component and the squamous marker P63 was detected in the squamous cell carcinoma (SCC) component (Fig. 5)**.** Postoperative outcome was uneventful. The patient was discharged two weeks after surgery and experiences no tumor recurrence or metastasis after a 6-month follow-up period.

## Discussion

Most malignancies of the ampulla of Vater are adenocarcinomas while other histopathological type such as adenosquamous carcinoma (ASC) is extremely rare [1]. ASC has two histologically malignant components, including adenocarcinoma and squamous cell carcinoma (SCC). It was first reported in 1907 [2]. Although the histogenesis of ASC remains uncertain, ASC is considered to have even worse prognosis and clinically more aggressive behavior than adenocarcinoma [3]. ASCs are usually located in organs where adenocarcinomas and squamous cell carcinomas respectively predominate, such as intestine [4], stomach [5], esophagus [6] and vagina [7]. ASCs in the above primary sites are also exceedingly rare.

In consideration of the rarity of ASC of the AmV, its oncogenic mechanisms, overall prognosis and therapeutic strategies remain largely unclear. Four theories have been hypothesized as the possible histogenesis of ASCs: collision of both squamous tumor and adenocarcinoma; squamous metaplasia; adenocarcinoma transforming into squamous cell carcinoma; pluripotent stem cells origin [8].

We systematically searched the online literature databases Wanfang, Emabase, Pubmed, Medline, Cochrane Collaboration Library and China National Knowledge Infrastructure (CNKI) for articles using the terms “ampulla of Vater (AmV)”and “adenosuqamous carcinoma (ASC)”. Eventually, 17 previously reported cases associated with ASC of the AmV were identified [2, 3, 9–15]. We have summarized the patient demographics, clinical investigations, operative methods, histopathological analyses, postoperative treatment and overall prognosis of the identified reports including the present case in Table 1.

**Table 1:** Clinical parameters, management and prognosis of reported cases with adenosquamous carcinoma of the ampulla of Vater

	Year	Author	Age	Gender	Symptom	Examination	Size (mm)	Preoperative biopsy	Operative method	LN metastasis	Postoperative radiation or chemotherapy	Stage	Prognosis (months)	Postoperative distant metastasis
1	2002	Ueno et al.[10]	47	M	Jaundice, fatigue	ERCP, duodenoscopy	22	SCC	PD	(−)	ND	IV	10 Dead	( +)
2	2005	Ri et al.[11]	62	F	Jaundice, fatigue, abdominal pain	Contrast-enhanced CT, duodenoscopy, ERCP	15	ND	PPPD	(−)	( +)	II	11 Dead	( +)
3	2013	Yang et al.[3]	64	M	Jaundice, abdominal pain	Contrast-enhanced CT, ERCP	34	ADC	PD	( +)	( +)	IIB	6 Dead	( +)
4	2013	Yang et al.[3]	82	M	Jaundice	NM	NM	ND	Ampullectomy	(−)	ND	IB	14 Dead	( +)
5	2013	Yang et al.[3]	68	M	Jaundice, abdominal pain	NM	NM	SCC	PD	( +)	NM	III	7 Dead	( +)
6	2013	Yang et al.[3]	34	F	Jaundice, abdominal pain	NM	NM	SCC	PD	(−)	NM	III	10 Dead	( +)
7	2013	Yang et al.[3]	77	M	Jaundice, abdominal pain	NM	NM	SCC	PD	(−)	NM	IIB	6 Dead	( +)
8	2014	Kshirsagar et al. [2]	58	M	Jaundice, abdominal pain, vomiting	Contrast-enhanced CT, ERCP	40	SCC	ERCP guided stent	(−)	ND	NM	NM	NM
9	2015	Hoshimoto et al. [9]	81	F	No symptoms	Contrast-enhanced CT, MRCP, endoscopic ultrasonography, duodenoscopy, ERCP	11	ADC + SCC	PPPD	(−)	ND	IA	20 Alive	(-)
10	2018	Carvalho et al.[12]	68	M	Jaundice, choluria, acholia	Endoscopic ultrasonography	NM	ADC	PD	( +)	ND	IIIA	10 Alive	(-)
11	2018	Milanetto et al.[13]	81	F	Jaundice, pruritus, hyperchromic urine, acholic stool	Contrast-enhanced CT, abdominal ultrasonography	40	ND	PPPD	( +)	ND	NM	16 Dead	( +)
12	2019	Kun Wang et al. [14]	60	M	Jaundice, fatigue, hyperchromic urine	Contrast-enhanced CT	25	ND	PD	(−)	ND	IIA	8 Alive	(-)
13	2014	Xue Wen et al. [15]	59	F	Jaundice, abdominal pain	NM	46	ND	PD	( +)	ND	III	7 Dead	NM
14	2014	Xue Wen et al. [15]	59	F	Jaundice	NM	35	ND	PD	( +)	( +)	III	11 Dead	NM
15	2014	Xue Wen et al. [15]	67	M	Jaundice, abdominal distension	NM	22	ND	PD	( +)	ND	II	2 Dead	NM
16	2014	Xue Wen et al. [15]	54	M	Jaundice	NM	15	ND	PD	(−)	ND	I	10 Dead	NM
17	2014	Xue Wen et al. [15]	66	M	Jaundice, abdominal pain	NM	27	ND	PD	(−)	ND	III	8 Dead	NM
18		Present case	67	F	Jaundice, pruritus, hyperchromic urine, acholic stool, vomiting	Contrast-enhanced CT, MRCP, ERCP, PET/CT	36	ADC	laparoscopic radical gastrectomy + PD	(−)	ND	IB	6 Alive	(-)

*ND* not done, *NM* not mentioned, *SCC* squamous cell carcinoma, *ADC* adenocarcinoma, *PD* pancreaticoduodenectomy, *PPPD* pylorus-preserving pancreaticoduodenectomy, *CT* computed tomography, *PET/CT* positron emission tomography–computed tomography, *MRCP* Magnetic Resonance Cholangiopancreatography, *ERCP* Endoscopic Retrograde Cholangiopancreatography

The average age at clinical diagnosis was 64.1 years old (range: 34 to 82) including 11 of 18 patients (61.2%) were men. Almost all of the cases presented with jaundice and other presenting symptoms included adnominal pain (8/18, 44.4%), hyperchromic urine (4/18, 22.2%) and acholic stool (3/18, 16.67%). In the review of the identified 18 cases (including the present case) of ASC AV, 8 cases revealed the laboratory investigations of which 6 patents (6/8, 75.0%) had elevated total bilirubin levels and 8 patients (8/8, 100%) had elevated hepatic and biliary enzymes. Serum tumor markers such as carbohydrate antigen 19–9 (CA 19–9) and carcinoembryonic antigen (CEA) were measured preoperatively in 7 cases. And elevated serum CA 19–9 and/ or CEA levels were identified in 4 (4/7, 57.1%) cases.

9 cases mentioned the preoperative radiological methods including abdominal computed tomography (CT) (7/9, 77.8%), endoscopic retrograde cholangiopancreatography (ERCP) (6/9, 66.7%), duodenoscopy (3/9, 33.3%), magnetic resonance cholangiopancreatography (MRCP) (2/9, 22.2%), endoscopic ultrasonography (2/9, 22.2%) and positron emission tomography–computed tomography (PET/CT) (1/9, 11.1%). Most of the reported cases of ASC of the AmV underwent abdominal CT and ERCP which indicated that abdominal CT and ERCP are valuable and effective for achieving the preoperative diagnosis. In the present case, PET/CT also played an important role in preoperative diagnosis revealing an increased metabolic focus at the AmV. Some studies mention that PET/CT could be used to detect post-operational early recurrence and distal metastasis while more evidence is still needed to confirm its efficacy [2].

Some authors suggest that for patients who can be diagnosed with the ASC of AmV before surgery, conservative treatment such as chemoradiation is strongly recommended to serve as the most appropriate therapeutic strategy [16]. However, it is difficult to draw firm conclusions about its diagnosis by preoperative limited histologic evaluations because of the difficulty in acquiring both adenocarcinoma and squamous cell carcinoma components [3]. Although 9 patients underwent the preoperative fine needle tumor biopsy, only one case of them (1/9, 11.1%) exhibited patterns typical of both malignant SCC and ADC components before surgical resection which also suggests the difficulty in acquiring both ADC and SCC components in ADC patients prior to surgeries. Thus, it is actually difficult to perform conservative treatment such as chemoradiation on patients with ASC of the AmV because of the difficulty of determining the correct preoperative histologic diagnosis. And surgical interventions still remain to be the first choice therapy for patients with ASC of the AmV detected at an early stage [9, 17].

17 reported patients with ASC of the AmV underwent surgical treatment including pancreaticoduodenectomy (13/17, 76.5%), pylorus-preserving pancreaticoduodenectomy (3/17, 17.6%) and ampullectomy (1/17, 5.9%). Although most of the studies still recommend surgical resections as the best therapy for patients with ASC of the AmV at an early stage [9, 17], the limited clinicopathological perception of the disease makes it difficult to draw the firm conclusion on the efficacy and benefits of performing surgeries in these patients based on the high morbidity of the surgeries. Thus, further studies in larger cohorts are needed to elucidate the therapeutic strategies for patients with ASC of the AmV.

12 cases reported the tumor size. The tumor size in 12 cases ranged from 11 to 46 mm with a median of 27.67 mm. Lymph node metastases were detected in 7 of 18 patients (38.9%). Postoperative chemotherapy and radiotherapy were performed in 3 cases. 16 of all 18 cases have reported the tumor stage including I in 4 patients, II in 5, III in 6 and IV in 1.

In the review of 18 cases, 12 of them have studied the postoperative distal metastasis during the follow-up period. And postoperative distant metastasis was identified in 67.7% (8/12) of the reported patients with ASC of the AmV which is consistent with the acknowledged conclusion that ASC of the AmV has high possibility of early distal metastasis after surgery resection [2]. The median time of follow-up was 9.5 months ranging from 2 to 20 months. 13 patients (72.2%) died within 16 months after surgical resection and their median survival was 9.08 months which also demonstrates that ASC of the AmV has unfavorable prognosis and short survival. However, further studies are needed to compare the overall prognosis between ASC of the AmV and conventional adenocarcinoma of the AmV and to elucidate the relationship between the prognosis of patients with ASC of the AmV and the proportion of SCC components. Several studies suggest that the proportion of SCC components in the ASC of the AmV might be positively correlated with disease progression [18, 19]. Whether Ki-67 index of the SCC and ADC components indicate their proliferative potential and are associated with the overall progression and clinicopathologic characteristics in patients with ASC of the AmV still warrant further investigation.

Of note, the frequency of simultaneous multiple primary cancers of the AmV and stomach is exceedingly low, only four cases are available [20–22]. H. Yamashita et al. [20] once reported 2 cases both of which had adenocarcinoma of the AmV coexistent with gastric adenocarcinoma. Prior to H. Yamashita’s report, only one case regarding multiple primary cancers of the AmV and stomach is available in the English literature [21]. Masahide Fukaya et al. [22] once reported a case of esophageal squamous cell carcinoma, gastric adenocarcinoma with synchronous occurrence of the small cell carcinoma at the AmV who underwent a subtotal esophagectomy and gastrostomy as the first-stage operation and a total gastrectomy and a PD as the second-stage surgery. However, to the best of our knowledge, the finding of poorly differentiated gastric adenocarcinoma which achieved complete response to neoadjuvant therapy synchronously associated with ASC of the AmV has never been reported in the literature.

More frequent follow-up after surgery and a better understanding of its clinicopathological features are necessary for detecting possible early recurrence and distal metastasis and providing practical therapeutic strategies in patients with ASC of the AmV.

## Conclusions

Adenosquamous carcinoma of the ampulla of Vater (ASC of the AmV) containing both adenocarcinoma and squamous cell carcinoma components are exceedingly rare. Furthermore, to the best of our knowledge, this is the first report of coexistent gastric adenocarcinoma which achieved complete remission after chemotherapy with ASC of the AmV. The relationship of gastric adenocarcinoma and ASC of the AmV in etiology and pathogenesis still remains unelucidated and requires further investigation. And more studies should be done to determine the clinical progression and the best treatment methods in patients of ASC of the AmV coexistent with gastric carcinoma.

## Data Availability

All patient data and clinical images adopted are contained in the medical files of Peking Union Medical College Hospital. The data supporting the conclusions of this article is included within the manuscript, figures and tables.

## Abbreviations

ASC: Adenosquamous carcinoma;
AmV: Ampulla of Vater
CT: Computed tomography
PET/CT: Positron emission tomography/computed tomography
MRCP: Magnetic resonance cholangiopancreatography
ERCP: Endoscopic retrograde cholangiopancreatography
SCC: Squamous cell carcinoma
ADC: Adenocarcinoma
CA 19–9: Carbohydrate antigen 19–9
CEA: Carcinoembryonic antigen
PD: Pancreaticoduodenectomy
PPPD: Pylorus-preserving pancreaticoduodenectomy
